# Shaping Polarization Of Tumor-Associated Macrophages In Cancer Immunotherapy

**DOI:** 10.3389/fimmu.2022.888713

**Published:** 2022-06-30

**Authors:** Jing Gao, Yuanzheng Liang, Liang Wang

**Affiliations:** ^1^Beijing Tongren Hospital, Capital Medical University, Beijing, China; ^2^Department of Hematology, Beijing Tongren Hospital, Capital Medical University, Beijing, China

**Keywords:** tumor-associated macrophages, polarization, tumor microenvironment, signaling pathways, cancer therapy

## Abstract

Different stimuli can polarize macrophages into two basic types, M1 and M2. Tumor-associated macrophages (TAMs) in the tumor microenvironment (TME) are composed of heterogeneous subpopulations, which include the M1 anti-tumor and M2 pro-tumor phenotypes. TAMs predominantly play a M2-like tumor-promoting role in the TME and regulate various malignant effects, such as angiogenesis, immune suppression, and tumor metastasis; hence, TAMs have emerged as a hot topic of research in cancer therapy. This review focuses on three main aspects of TAMs. First, we summarize macrophage polarization along with the effects on the TME. Second, recent advances and challenges in cancer treatment and the role of M2-like TAMs in immune checkpoint blockade and CAR-T cell therapy are emphasized. Finally, factors, such as signaling pathways, associated with TAM polarization and potential strategies for targeting TAM repolarization to the M1 pro-inflammatory phenotype for cancer therapy are discussed.

## 1 Introduction

Macrophages are an important part of the mononuclear phagocytic system and are involved in immune system regulation, pathogen clearance, wound healing, and angiogenesis. Furthermore, there is a close relationship between macrophages and tumors. Under the stimulation of various cytokines, macrophages can be polarized into two forms that exhibit different functions: M1 macrophages, which are pro-inflammatory and tumor-inhibiting, and M2 macrophages, which are anti-inflammatory and tumor-supporting. Therefore, macrophages that infiltrate the tumor microenvironment (TME), also known as tumor-associated macrophages (TAMs), have gradually attracted attention. TAMs are generally M2-like anti-inflammatory immune cells and are associated with malignant disease progression, drug resistance, and poor prognosis. Current cancer treatment strategies are not limited to traditional radiotherapy, chemotherapy, or surgical resection as cancer treatment has entered the era of targeted therapy and immunotherapy. Modulation of TAMs by regulating M1 signaling activation has emerged as a promising and novel immunotherapy strategy. Hence, understanding the role of signaling pathways associated with TAM polarization and approaches that can regulate TAM repolarization provide a new perspective for cancer therapy.

## 2 Polarization of TAMs and Their Role in the TME

TAMs are a specific group of macrophages that reside in the TME. Stimulated by different factors, these macrophages exhibit different phenotypes and functions, through a process termed as TAM polarization. Understanding the cellular and molecular mechanisms associated with TAM polarization in the TME contributes to a deeper insight into tumor pathogenesis and can provide new insights for cancer therapy. In this section, we focus on TAM polarization and their role in the TME.

### 2.1 Differentiation of Macrophages

**Figure 1:**
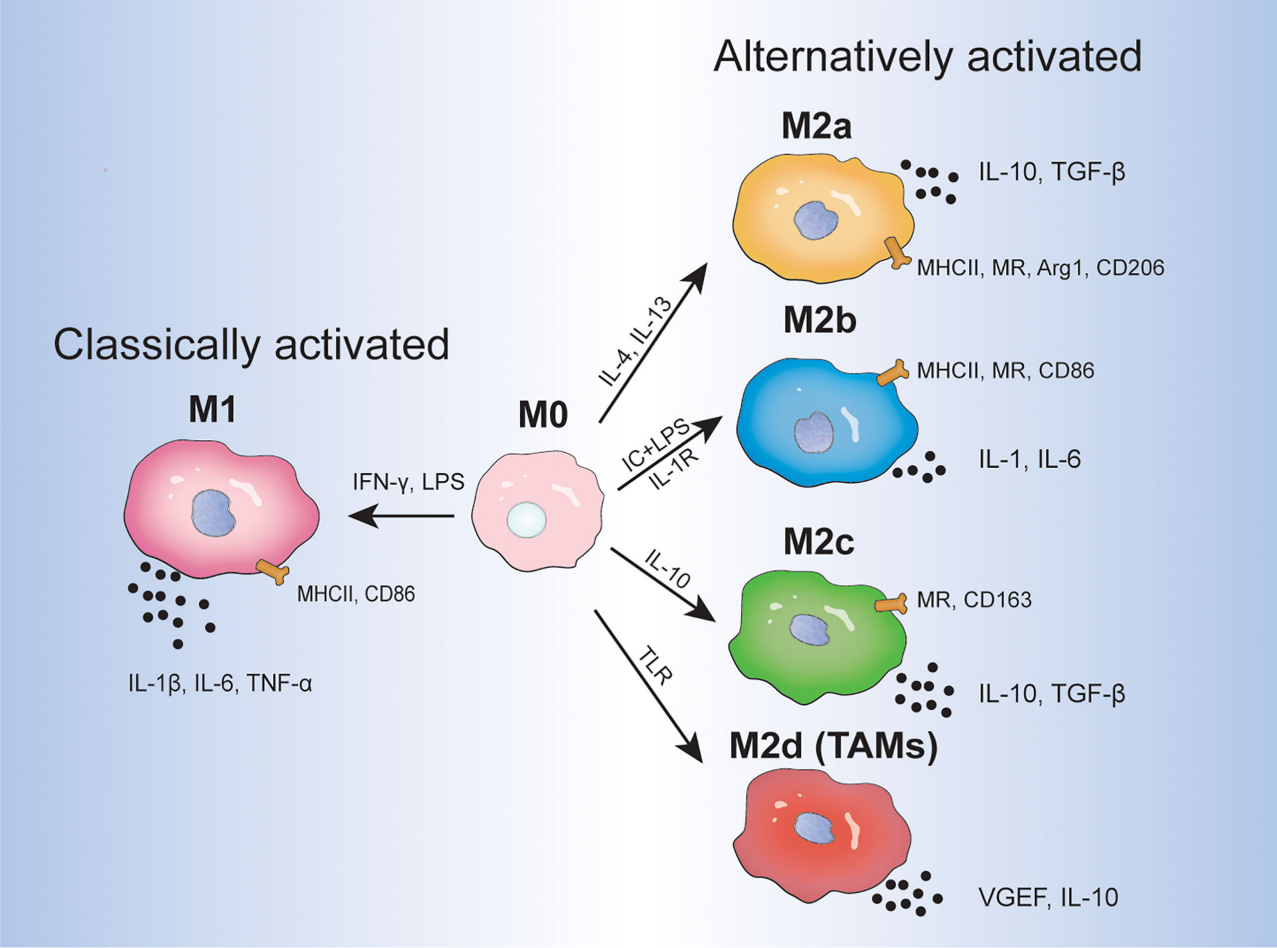
The direction of macrophage differentiation in response to different environmental cues. IC, immune complex; LPS, lipopolysaccharide; TLR, Toll-like receptor; VEGF, vascular endothelial growth factor; MR, mannose receptor; Arg 1, arginase 1.

Macrophages are involved in host defense, wound healing, and immune regulation and differentiate into different phenotypes in response to environmental cues (1). Due to the plasticity of macrophages, undifferentiated macrophages (M0) can be polarized into two types: classically activated macrophages (M1) and alternatively activated macrophages (M2) (2). M1 macrophages, which are stimulated by interferon (IFN)-γ (produced by T-helper 1 cells) and bacterial lipopolysaccharide, are generally considered to have pro-inflammatory and anti-tumor effects and express inflammatory factors including interleukin (IL)-1β, IL-6, and tumor necrosis factor (TNF)-α. In contrast, M2 macrophages, which are stimulated by IL-4 and IL-13 (produced by T-helper 2 cells), play a critical role in tumor initiation, proliferation, metastasis, and immune evasion, and express anti-inflammatory elements, such as IL-10 and transforming growth factor (TGF)-β (3, 4). It is worth noting that macrophages are a heterogeneous group of immune cells and are not just classified into M1 and M2 macrophages. Mantovani et al. further classified activated M2 macrophages into M2a, M2b, and M2c macrophages, which are stimulated by IL-4/IL-13, immune complexes and lipopolysaccharide/IL-1 receptor, and IL-10, respectively (5). M2a and M2b macrophages play an immunomodulatory role and promote T-helper 2 cell response, whereas M2c macrophages are associated with immune response suppression and tissue remodeling. Additionally, the concept of M2d macrophages (also at times termed as TAMs) that are activated by Toll-like receptors and specifically express vascular endothelial growth factor (VEGF) and IL-10 was proposed (2, 4). Functionally, M2d macrophages participate in angiogenesis and tumor progression. Different inducers, surface markers, and cytokine products are shown in **Figure 1**. Given that the differential activation of macrophages can promote or inhibit inflammation as well as regulate tumor proliferation, targeting TAMs in the TME is receiving increasing attention.

### 2.2 TME and TAM Polarization

The TME is a highly complex and critical environment that is encased on the outside by collagen and elastin fibers and is composed of blood vessels, cancer cells, stromal cells, and immune cells, such as T cells, B cells, dendritic cells, myeloid-derived suppressor cells, and macrophages (6). The network formed by these elements participates in the recruitment of tumor and immune cells, constructing a tumorigenic environment that promotes drug resistance. Thus far, various mechanisms and pathways involved in immune modulation, angiogenesis, and metastasis have been studied to gain a deeper understanding of the interactions between these components in the TME. For example, cytokines in the TME, such as TNF-α, IL-6, and IL-8, are associated with angiogenesis and tumor metastasis, whereas IL-4, IL-13, and IL-10 are associated with immune response suppression. Moreover, various signaling pathways associated with macrophage polarization, including nuclear factor kappa B (NF-κB) and signal transducer and activator of transcription 1 (STAT1), are also relevant to the TME and are discussed below (5). In this context, identifying and targeting immunosuppressive elements in the TME may shed light on the mechanisms underlying tumor generation and development.

TAMs are one of the most common immune cells that infiltrate the TME. These cells originate from two main sources: bone marrow peripheral monocytes and embryos that reside in different tissues, the latter including Kupffer cells in the liver, alveolar macrophages in the lungs, microglia in the brain, and osteoclasts in the bone (7). Peripheral blood circulating monocytes, which are recruited into the TME by circulating tumor-secreting factors and transform into macrophages, are generally thought to be the main source of TAMs. In contrast, a small number of macrophages are derived from early tissue-resident macrophages originating in the yolk sac or fetal liver (8). Broadly speaking, monocytes are attracted by cytokines, such as colony stimulating factor (CSF)-1 and CCL-2, and subsequently polarize into TAMs in the TME. These polarized TAMs usually express M2 macrophage markers and cytokines, such as mannose receptors (CD206), scavenger receptor (CD163), VEGF, and IL-10, and exhibit tumor-supporting effects, and are hence called M2-like TAMs. Conversely, few TAMs in the TME express CD86 and CD80 markers and are termed as M1-like TAMs, and typically exhibit anti-tumor effects (9).

### 2.3 Role of M1-Like TAMs in the TME

The role of M1-like macrophages in the TME is mainly pro-inflammatory and they inhibit tumor progression. Most studies have focused on M2-like macrophages, which play a pro-tumor role and constitute the predominant class of TAMs. However, some researchers have suggested that the role of M1-like TAMs in tumors is bidirectional. It has been demonstrated that CD68+ HLA-DR+ M1-like TAMs enhance the motility of tumor cells in hepatocellular carcinoma. Additionally, exosomes in oral squamous cell carcinoma have been reported to regulate TAM conversion to M1-like TAMs, which subsequently promotes malignant tumor metastasis (10, 11). The association of M1-like macrophages with tumor metastasis may be partly due to the effects of inflammatory cytokines, such as IL-1β, TNF-α, and IL-6, which directly or indirectly contribute to vasoproliferation (12, 13). Therefore, the multifaceted role of inflammatory factors in TME requires further study.

### 2.4 Role of M2-Like TAMs in the TME

**Figure 2:**
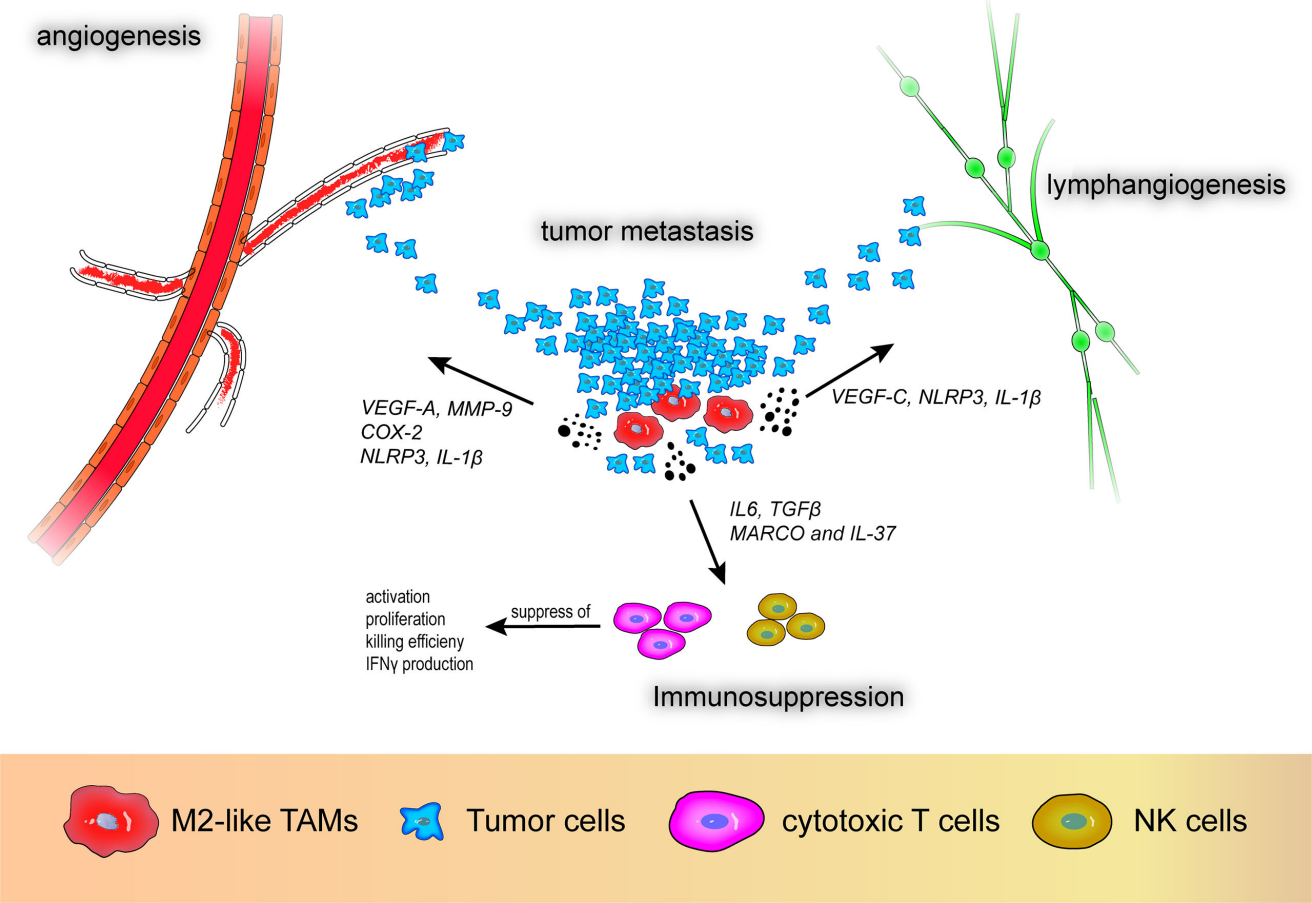
M2-like tumor-associated macrophages promote angiogenesis, lymphangiogenesis, immunosuppression, and tumor metastasis. VEGF-A, vascular endothelial growth factor A; VEGF-C, vascular endothelial growth factor C; MMP-9, matrix metalloproteinase 9; COX-2, cyclooxygenase-2; MARCO, macrophage receptor with collagenous structure; NLRP3, nod-like receptor protein 3.

The role of M2-like macrophages in the TME is mainly anti-inflammatory and they promote tumor progression. Based on the various surface markers and cytokines expressed by M2-like TAMs, their pro-tumor effects can be divided into three aspects: angiogenesis, immunosuppression, and metastasis. M2-like TAMs secrete growth factors (VEGF, platelet-derived growth factor, epidermal growth factor, TGF-β), matrix metalloproteinases (MMPs) (MMP-2, MMP-9), and other cytokines (TNF-α, IL-1β, IL-8) that are pro-angiogenic and promote metastasis as well as inhibit T and natural killer (NK) cells leading to an attenuated immune response (14, 15). In addition, other factors produced by M2-like TAMs, such as heme oxygenase-1 (HO-1) and cyclooxygenase-2 (COX-2), are involved in carcinogenesis and angiogenesis through different pathways. The roles of M2-like TAMs in the TME are shown in **Figure 2**.

With respect to vascular generation and metastasis, VEGF-A participates in tumor angiogenesis and research has demonstrated that high expression of VEGF-A in M2-like macrophages promotes angiogenesis in patients with non-small cell lung cancer (NSCLC), which is associated with poor prognosis (16). Additionally, MMPs also play a pivotal role in tumor angiogenesis. Wu et al. reported that CXCL-8 derived from TAM-like peripheral blood monocyte-derived macrophages promote secretion of MMP-9 in bladder cancer cells, subsequently causing alterations in the migration, aggression, and proangiogenic capacity of bladder tumor cells (17). Moreover, HO-1 and COX-2 secreted by M2-like TAMs can promote tumor growth. HO-1, a stress-responsive oxidative inflammatory protein, is regulated by IL-10, nuclear factor-erythroid factor 2-related factor 2, and Bach1 and is a potential immunomodulatory target (18). Arnold et al. reported that macrophages with high expression of fibroblast activation protein-α and F4/80 in the LL2/OVA cancer cell line shows M2 an like malignancy and were associated with increased expression of HO-1, which is related to tumor immune suppression (19). Furthermore, Kim et al. demonstrated that paclitaxel-induced tumor cell debris can induce expression of HO-1 in macrophages, resulting in an anti-tumor immune response and dampened efficacy of paclitaxel in breast cancer (20). COX-2 is involved in inflammatory processes and tumor invasion (21). Moreover, prostaglandin E2 and thromboxane A2, downstream products of COX-2-expressing macrophages, can promote angiogenesis and disrupt immune cell function in solid tumors and COX-2 inhibitors can reverse this effect (12, 22). In addition to angiogenesis and metastasis, the immunosuppressive effect of M2-like macrophages, i.e., inhibition of T and NK cell activity and proliferation, contributes to the malignant progression of tumors. La Fleur et al. reported positive expression of macrophage receptor with collagenous structure (MARCO) and IL-37 in IL-4- and IL-10-stimulated depolarized macrophages that exhibit M2-like effects in NSCLC (23). The authors suggested that MARCO+ M2-like TAMs are not only associated with a robust anti-inflammatory environment, but can also diminish anti-tumor immunity through various mechanisms including suppression of T cell activation, proliferation, and killing efficiency, decreased IFN-γ production, and inhibition of NK cell function.

Recently, TAMs have been reported to promote lymphangiogenesis in tumors. Hwang et al. reported that high VEGF-C expression in M2-like TAMs promotes lymphangiogenesis (16). Moreover, Weichand et al. reported the essential role of the sphingosine-1-phosphate receptor 1 signaling pathway and its downstream secretory molecules NOD-like receptor protein 3 and IL-1β in metastasis, angiogenesis, and lymphangiogenesis in breast cancer (24). Using *in vitro* experiments and dataset analysis, the authors also demonstrated that through this pathway, IL-1β is related to lymphatic vessel proliferation, whereas NOD-like receptor protein 3 is related to breast cancer invasion and metastasis. Due to the polymorphisms of TAMs and the complexity of related molecules, the interaction between TAMs and the TME in tumors requires further study.

## 3 Cancer Immunotherapy and M2-Like TAMs

Given that traditional cancer treatment strategies, such as radiotherapy, chemotherapy, and surgical excision, are associated with challenges of resistance and recurrence, a variety of immune checkpoint and checkpoint blockade immunotherapy strategies have been proposed and are starting to shed light on the treatment of various cancer types. These immune checkpoint-associated therapies, also known as immune checkpoint blockade (ICB) or immune checkpoint inhibitors (ICIs), include targeting and antagonizing cytotoxic T-lymphocyte-associated protein 4 (CTLA-4) and programmed cell death protein 1 (PD-1), and its ligands (programmed death ligand 1 [PD-L1] and 2 [PD-L2]) (25). Moreover, chimeric antigen receptor (CAR)-T cell therapy is another immunotherapy strategy that has achieved tremendous breakthrough in recent years and is mainly applied in non-solid tumors, such as leukemia and lymphoma (26). TAMs are essential components of the TME and play a prominent role in these therapeutic processes and pave the way for the creation of new therapeutic approaches. In this section, recent advances and challenges in cancer treatment as well as the role of M2-like TAMs in ICB and CAR-T cell therapy are discussed.

### 3.1 Recent Advances and Challenges in Cancer Treatment

CTLA-4 is a receptor located on the surface of T cells that dampens T cell activity and promotes tumor proliferation. Mechanistically, CTLA-4 prevents uncontrolled expansion of activated T cells by competitively binding to CD80/CD86 receptors on CD28-expressing dendritic cells. Similarly, PD-1 is another immune checkpoint receptor that is often expressed on the surface of tumor-infiltrating lymphocytes, while its ligands PD-L1 and PD-L2 are highly expressed on tumors; the interaction of PD-1 with PD-L1/PD-L2 can lead to a diminished immune response (25). Hence, blocking immune checkpoints, such as CTLA-4, PD-1, and PD-L1/PD-L2, using ICB drugs has become a widely investigated strategy for cancer treatment. The CTLA-4 antibody ipilimumab and anti-PD-1 antibodies, pembrolizumab and nivolumab, have been approved by the United States Food and Drug Administration as therapeutic agents for patients with melanoma (27). PD-L1 antibodies durvalumab and avelumab have also been approved for use in different cancers. However, despite the unprecedented success of ICB, its efficacy against “cold” tumors, such as glioblastoma (GBM), remains elusive, in part due to TIM-3 upregulation and the blocking effect of the blood–brain barrier (28). In addition, due to a multitude of host endogenous or exogenous factors, the therapeutic response to ICB in cancer is often restricted, either effective only in specific tumor types or in selected patients. Furthermore, the prevalence of immune-related adverse events associated with ICB therapy remains high and the underlying mechanisms remain unclear and require further study.

Presently, CARs can be divided into five generations: the first generation, which expresses the basic CD3ζ signal; the second and third generations, which express expressing co-stimulatory domains; the fourth generation, which expresses cytokine-expression domains; and the fifth generation, which expresses cytokine receptor-expressing domains (28). There are four generations of CAR-T cell therapy, and the current United States Food and Drug Administration-approved products are mainly second-generation CAR-T cells with CD28 or 4-1BB co-stimulatory receptors. The process of CAR-T cell therapy is as follows: 1) isolation of leukocytes from patients; 2) *in vitro* activation of T cells by T-cell receptor and CD28; 3) introduction of CARs using viral or non-viral vectors; 4) culture and proliferation of CAR-T cells; and 5) transfusion of amplified CAR-T cells to the patient (26). However, a variety of factors, such as antigen mutations, CAR-T cell depletion, and suppressive TME, are thought to be associated with resistance. For example, the absence of antigens, especially CD19 mutations, is an important cause of CAR-T cell therapy failure, while prolonged CAR activation leads to apoptosis and T cell failure. Additionally, the immunosuppressive effect exerted by cytokines, such as prostaglandin E2, IL-6, IL-10, and TGF-β in the TME, as well as infiltrating regulatory T cells and TAMs, are obstacles to CAR-T cell therapy (29). Moreover, although CAR-T cell therapy has shown promising results in hematologic diseases, such as leukemia and lymphoma, it has not been as effective in the treatment of solid tumors, such as GBM (14, 28, 29).

### 3.2 Role of M2-Like TAMs in ICB and CAR-T Cell Therapy

The application of ICB drugs and the novel concept of harnessing the “CAR” devices to active and direct T cells has brought a considerable breakthrough in the field of oncology. Given that the limitations of these two therapeutic approaches exist primarily in solid tumors rather than in hematologic tumors, a better understanding of the mechanisms underlying resistance in solid tumors may help improve the modalities and efficacy of immunotherapy. With this regard, the activity of M2-like TAMs in the TME is highly correlated with immune downregulation and resistance to these treatments, which needs to be urgently addressed.

M2-like TAMs induce resistance to ICB therapy in solid tumors, the underlying mechanisms include, but are not limited to, recruitment to the TME *via* tumor-derived chemokines, such as CXCL8 and CCL2, to mediate local immunosuppression, as well as the direct expression of high levels of PD-L2 but not PD-L1 to escape anti-PD-1 targeted therapies (30, 31). For example, immunosuppressive factors, such as arginase-1, secreted by CXCR2+CD68+ macrophages that infiltrate the TME, lead to functional failure of T cells and tolerance to ICB. Mingjie et al. suggested that IFN-γ could inhibit CXCL8 secretion in pancreatic cancer and prevent the recruitment of CXCR2+CD68+ macrophages, thereby improving the efficacy of anti-PD-1 therapies (30). Additionally, PD-L2 exerts immunosuppressive effects similar to PD-L1 but is not sensitive to anti-PD-L1 drugs, and the expression of PD-L2 on M2-like TAMs can also lead to therapeutic failure. Yang et al. demonstrated that blockade of the CCL2–CCR2 axis in esophageal squamous cell carcinoma contributes to the downregulation of infiltrating TAMs, thereby reducing PD-L2 expression and inhibiting immunosuppression (31). In summary, chemokines that recruit TAMs into the TME and the variable expression of immune checkpoints on M2-like TAMs contribute to resistance to ICB. Furthermore, upon interaction with other factors, such as progranulin (PGRN) and lipid accumulation, TAMs can suppress immune responses with PD-L1 overexpression (32, 33). PGRN is expressed in inflammatory diseases and tumors and is implicated in neurodegeneration, tissue damage repair, and embryonic development. Fang et al. demonstrated that PGRN upregulates the expression of PD-L1 and M2-related receptors on TAMs by stimulating the Janus kinase (JAK)/STAT3 signaling pathway in breast cancer (32). Similarly, Qin Luo et al. reported that lipid accumulation enhances the expression of M2-associated markers (CD206 and CD11c) and PD-L1 on TAMs through the phosphoinositide 3-kinase (PI3K)-γ signaling pathway in gastric cancer (33). The findings of these studies suggest that signaling pathways associated with M2-like TAMs may contribute to resistance to ICB therapy and poor prognosis by modulating immune receptors, such as PD-1.

Moreover, poor performance of CAR-T cell therapy in solid tumors has put its development in a difficult position, and targeting M2-like TAMs in the TME is one potential approach for addressing this challenge. Recently, Rodriguez-Garcia et al. reported that a subpopulation of TAMs characterized by folate receptor beta (FRβ) can lead to antigen-specific T-cell dysfunction and failure of hMeso CAR-T cell therapy (34). The authors used various approaches, including gene expression analyses, flow cytometry, multiplex cytokine analysis, and oxygen consumption assays, to confirm that FRβ+ TAMs exhibit M2-like profiles. FRβ+ TAMs were found to express high levels of CD204, CD206, and IL-10 in mouse models of ovarian cancer, colon cancer, and melanoma, exhibited elongated cell shape, and displayed high oxygen consumption and metabolic capacity. Subsequently, they suggested that pretreatment of the TME with mFRβ CAR-T cell cells could arrest tumor expansion and enhance the potency of tumor-specific CAR-T cells, which provides a new direction to improve CAR-T cell therapy.

## 4 Targeting TAM Repolarization as a Cancer Treatment Strategy

Given that M2-like TAMs participate in various immunosuppressive processes in the TME, and also play a role in ICB and CAR-T cell therapy failure, strategies for targeting TAMs are attracting increasing attention. In general, targeting TAMs for cancer treatment has two main directions: 1) to prevent macrophage recruitment to the TME; and 2) to regulate TAM repolarization (14). Many attempts have been made to restrict the infiltration of TAMs by blocking the CSF-1/CSF-1 receptor and CCL2/CCR2 pathways with varying degrees of success (3). Furthermore, there has been wide interest in regulating the functions of TAMs. In this section, we review signaling pathways and other factors related to TAM reprogramming and discuss approaches for targeting TAM repolarization.

### 4.1 Signaling Pathways and TAMs Reprogramming

The pivotal role of TAMs in tumor evolution is associated with various signaling pathways, including, but not limited to, the STAT family, PI3Kγ/AKT and NF-κB signaling pathways, and other related pathways, such as the CD47/SIRPα signaling pathway (3, 9). Understanding the significance of each pathway in macrophage polarization is of great importance and provides insights into developing strategies that can regulate the conversion of M2-like TAMs to M1-like TAMs. Therefore, in the below section, we discuss several important signaling pathways associated with the repolarization of TAMs, and the relevant literature is presented in **Table 1**.

**Table 1:** Overview of several signaling pathways associated with the repolarization of tumor-associated macrophages.

Author	Disease	Year	Molecule	Mechanism	Function	References
Huffaker, T B et al.	melanoma	2021	IFNγ, NAMPT	Activate JAK/STAT1 signaling pathway	M1 polarization and better melanoma outcome	(35)
Kang Le et al.	Mantle cell lymphoma	2021	IL-10	Activate JAK/STAT1 signaling pathway	promote mantle cell lymphoma growth	(36)
Wenli Fang et al.	Breast cancer	2021	Progranulin	Activate JAK/STAT3 signaling pathway	M2 polarization and up-regulated the PD-L1 expression of TAMs	(32)
Qian Zhong et al.	Colorectal cancer	2020	CPEB3	Inhibit IL-6R/JAK/STAT3 signaling pathway	Attenuated tumor occurrence and inhibited CD163+ TAM polarization	(37)
Sifeng Tao et al.	Breast cancer	2020	linc00514	Activate STAT3/Jagged1/Notch signaling pathway	M2 polarization and breast cancer metastasis	(38)
Tao Yu et al.	/	2019	Trim24	STAT6 K383 acetylation	Restrain macrophage M2 polarization	(39)
Ying Wang et al.	Esophageal squamous cell carcinoma	2020	FOXO1	Activate FAK/PI3K/AKT signaling pathway	M2 polarization	(40)
Jason K. Sa et al.	Glioblastoma	2020	MARCO	Activate PI3Kγ/AKT signaling pathway, PTEN loss	M2 polarization and promote tumor growth	(41)
Man Li et al.	Pancreatic cancer	2020	BEZ	Inhibit PI3K/AKT signaling pathway	M1 polarization	(42)
Zhu et al.	Diffuse large B-cell lymphoma	2019	NSE	Enhance nuclear p50 translocation and inhibit NF-κB signaling pathway	M2 polarization and promotie the progression of lymphoma	(43)
Chia-Sing Lu	Non-small cell lung cancer	2020	JSH-23	Inhibit NF-κB signaling pathway	Restrain macrophage M2 polarization	(44)
Zhenxing Wang et al.	Non-small cell lung cancer	2020	CtBP1	Activate NF-κB signaling pathway	CCL2 secretion and M2 polarization	(45)
Marta Di Martile et al.	Melanoma	2020	Bcl-2	Activate NF-κB signaling pathway	Activation of IL-1β and M2 polarization	(46)
Michael Zhang et al.	Glioblastoma	2016	Anti-CD47 antibody	Inhibit CD47-SIRPα signaling pathway	M1 polarization	(47)

NAMPT, the rate limiting enzyme in NAD salvage synthesis; CPEB3, Cytoplasmic polyadenylation element binding protein 3; Trim24, a CBP-associated E3 ligase; FOXO1, The transcription factor forkhead box protein O1; MARCO, the macrophage receptor with collagenous structure; BEZ, PI3k-γ inhibitor; NSE, neuron-specific enolase.

#### 4.1.1 STAT Family Signaling Pathways

STAT family (STAT1, STAT2, STAT3, STAT4, STAT5A, STAT5B, and STAT6) plays an indispensable role in the efficiency of the mammalian immune system, and JAK (JAK1, JAK2, JAK3, and TYK2) is the most common upstream molecule of the STAT family (48). Both the JAK/STAT1 and JAK/STAT3 signaling pathways participate in macrophage polarization. The JAK/STAT1 signaling pathway can be activated by IFN-γ, which in turn upregulates the expression of nicotinamide phosphoribosyl transferase (NAMPT), the rate-limiting enzyme for NAD salvage synthesis (35). This study demonstrated that STAT1-induced NAMPT drives glycolysis, M1 polarization, and better outcomes in both mouse and human models of melanoma, while inhibition of NAMPT with FK866 reverses these effects. However, another study showed that IL-10 secreted by M2 macrophages can promote mantle cell lymphoma growth *via* the STAT1 signaling pathway (36). This suggests that STAT1 signaling pathway has complex roles and is involved in both M1 polarization that exerts pro-inflammatory effects and M2 polarization that exerts anti-inflammatory effects. The JAK/STAT3 signaling pathway is activated by various upstream molecules including PGRN and IL-6 in tumor models of breast and colorectal cancers, resulting in the induction of M2 macrophage polarization (32, 37). Qian et al. demonstrated that CPEB3, one of the four different CPEB-binding proteins, inhibits TAM-derived IL-6 and modulates CCL2 secretion, thus blocking this signaling pathway, attenuating tumor occurrence, and inhibiting CD163+ TAM polarization in colorectal cancer. Interestingly, studies have also reported that some long noncoding RNAs can bind directly to STAT3 rather than JAK and induce phosphorylation of STAT3 (49). For instance, Tao et al. demonstrated that long intergenic noncoding RNA 00514 regulates the expression of phosphorylated-STAT3 and activates the Jagged1-mediated Notch signaling pathway, leading to M2 polarization and breast cancer malignancy (38). STAT6 pathway is also known to drive M2 polarization. Trim24, a CREB-binding protein-associated E3 ligase, mediates CREB-binding protein ubiquitination at Lys119, and has been shown to promote STAT6 K383 acetylation and inhibits M2 polarization (39). Additionally, negative feedback by IL-4-activated STAT6 inhibits gene expression of *Trim24*, which may result in immunosuppressive profiles in the TME.

#### 4.1.2 PI3Kγ/AKT Signaling Pathway

Several studies have shown that the PI3Kγ/AKT signaling pathway is correlated with M2 polarization and poor prognosis in glioma, esophageal, gastric, and pancreatic cancers (40–42). Genomic analysis of GBM showed that mesenchymal-associated TAMs encoding high levels of MARCO play a role in the regulation of M2-like macrophages and accelerate tumor growth and aggressive phenotypes of GBM through loss of phosphatase and tensin homolog and activation of PI3Kγ/AKT signaling pathway (41). Similarly, in esophageal squamous cell carcinoma, forkhead box protein O1 promotes recruitment and polarization of M2 macrophages *via* the FAK/PI3K/AKT pathway, and the PI3K inhibitor LY294002 effectively suppresses the tumorigenic process of M2 macrophages (40). Additionally, the PI3Kγ pathway plays a major role in the formation of an immunosuppressive TME in pancreatic cancer, and its blocking drug BEZ successfully switched TAMs from M2 to M1 phenotype (42).

#### 4.1.3 NF-κB Signaling Pathway

Abnormal NF-κB activation is considered a contributing factor for tumor progression in some tumors, such as murine fibrosarcomas and diffuse large B-cell lymphoma; however, additional evidence suggests that it plays a protective role in promoting M2 differentiation and tumor progression (43, 50). For example, in NSCLC cells, the NF-κB inhibitor JSH-23 suppresses Oct4 expression, which subsequently inhibits the repolarization of monocytes into M2 macrophages (44). siRNA and BAY 11-7082 knock out P65 and impede p65 nuclear translocation, respectively, both of which block CtBP1-mediated CCL2 release by inhibiting the NF-κB signaling pathway (45). Similarly, in melanoma cells, the NF-κB repressor IKBα induces failure of Bcl-2-mediated M2 polarization (46). The complex role of the NF-κB pathway is partly due to its abundant downstream molecules, including chemokines, such as CCL2, as well as inflammatory cytokines, such as IL-1β, TNF-α, and IL-6 (45, 50). As mentioned previously, CCL2 is associated with monocyte recruitment and M2 polarization in the TME, whereas IL-1β, TNF-α, and IL-6 may play a dual role in tumors under different conditions.

#### 4.1.4 CD47/SIRPα Signaling Pathway

The CD47/SIRPα “do not eat me” signal is of great interest in terms of the anti-phagocytic ability of macrophages and researches targeting the CD47/SIRPα axis for better prognosis in various cancers such as ovarian, breast and colorectal cancer have also progressed today (51). Additionally, anti-CD47 treatment has been reported to regulate the transformation of M2-like TAMs into the M1 phenotype *in vivo* (47), suggesting a relationship between this pathway and reprogramming of macrophage polarization.

### 4.2 Other Factors Involved in Macrophage Polarization

#### 4.2.1 Lactic Acid and Tumor Acidosis Promotes M2 Macrophage Polarization

The 2019 Nobel Prize in Physiology or Medicine recognized a major discovery of the basic principles of how cells sense and respond to oxygen at the molecular level. Hypoxia-inducible factor 1α (HIF1α) has received increasing attention as an important component of the oxygen-sensing mechanism (52). The relationship between hypoxia, HIF1α, glycolysis, and lactic acid has been described previously. Interestingly, while M2 anti-inflammatory macrophages mainly rely on oxidative phosphorylation for energy, M1 pro-inflammatory macrophages rely mainly on glycolysis, which in turn increases lactic acid levels (53). It is worth noting that lactic acid facilitates M2 polarization through various mechanisms and some studies have shown that lactic acid driven from lactate dehydrogenase is responsible for tumor invasion. In pituitary adenoma, real-time quantitative reverse transcription PCR results indicated that high levels of lactate dehydrogenase A (*LDHA*) and *LAMP2* mRNA were associated with larger tumor size and stronger invasiveness. Subsequent cellular assays demonstrated that overproduction of lactic acid polarizes macrophages to the M2 phenotype, reshaping the TME (54). Additionally, bioinformatic analysis of 20 clinical samples of hepatocellular carcinoma revealed that overexpression of epithelial cell transformation sequence 2 leads to elevated lactate levels, resulting in polarization of M2 macrophages, a process that can be inhibited by knockdown of lactate dehydrogenase A (55). Tumor acidosis is another pro-tumor growth factor associated with glycolysis. For example, in melanoma, high level of glycolytic activity leads to accelerated glucose consumption and increased hydrogen ions (H+) and organic acids, which acidifies the TME and thus induces macrophages to express the transcriptional repressor ICER, eventually leads to the polarization of M2-like TAMs and subsequent immunosuppression. After excluding the effect of lactic acid, the authors demonstrated that the acidified environment in melanoma promotes non-inflammatory macrophage polarization and tumor growth (56). The association between the M1 and M2 macrophages is a dynamic process that is difficult to delineate. Therefore, it is crucial to regulate the balance between lactate levels and the degree of tumor acidosis in the TME to inhibit immunosuppression.

#### 4.2.2 Iron Promotes M1 Macrophage Polarization

Iron overload has received wide attention because of its functions in facilitating M1 polarization, inhibiting M2 activation, and exerting tumor immunotherapy effects. Mechanistically, iron overload polarizes macrophages to the M1 subtype through production of reactive oxygen species, thereby increasing p53 acetylation, which might participate in iron overload-induced macrophage polarization (57). Moreover, iron overload upregulates the expression of CCL2, IL-1β, TNF-α, and IL-6, suggesting activation of the NF-κB signaling pathway, thereby disrupting homeostasis of M1/M2 macrophage polarization (58). Since ionized iron can promote M1 macrophage polarization, Fe3O4 nanoparticles (NPs) were designed to target macrophage polarization and fight malignancy (59). It’s worth noting that Fe3O4 NPs are mainly internalized by macrophages and degraded to Fe2+ and Fe3+, thus playing a role in converting macrophage phenotypes to M1.

#### 4.2.3 Phytochemicals and Macrophages Polarization

Phytochemicals, which are extracted from natural plant products, also play an important role in mediating macrophage polarization and functions through various pathways. For example, curcumin was found to block M2 polarization of microglial cells in the mouse brain and increase the proportion of M1 polarization by inducing STAT1 and NF-kB signaling pathways, resulting in the decrease of GBM cells (60). Curcumin was also shown to recruit both NK cells and M1 macrophages into mouse GBM tumors, which reversed the immunosuppressed state in the GBM microenvironment (61). Similarly, rhodopsin, one of the main pharmacological active components of rhubarb, has also been found to have an inhibitory effect on tumor infiltration and M2 polarization. The mechanism may be the suppression of STAT6 and C/EBPβ signaling pathways (62). Furthermore, ginseng astragalus aqueous extract (WEGA) may also have the potential to become a new anti-cancer direction. Studies have revealed that WEGA can significantly inhibit the proliferation of human lung cancer cells A549 and stimulate the polarization of macrophages to M1 type (63). In conclusion, the application of phycocyanin, to a greater or lesser extent, is connected to the intrinsic mechanism of macrophage polarization and deserves further investigation.

### 4.3 Approaches for Targeting TAM Repolarization

**Figure 3:**
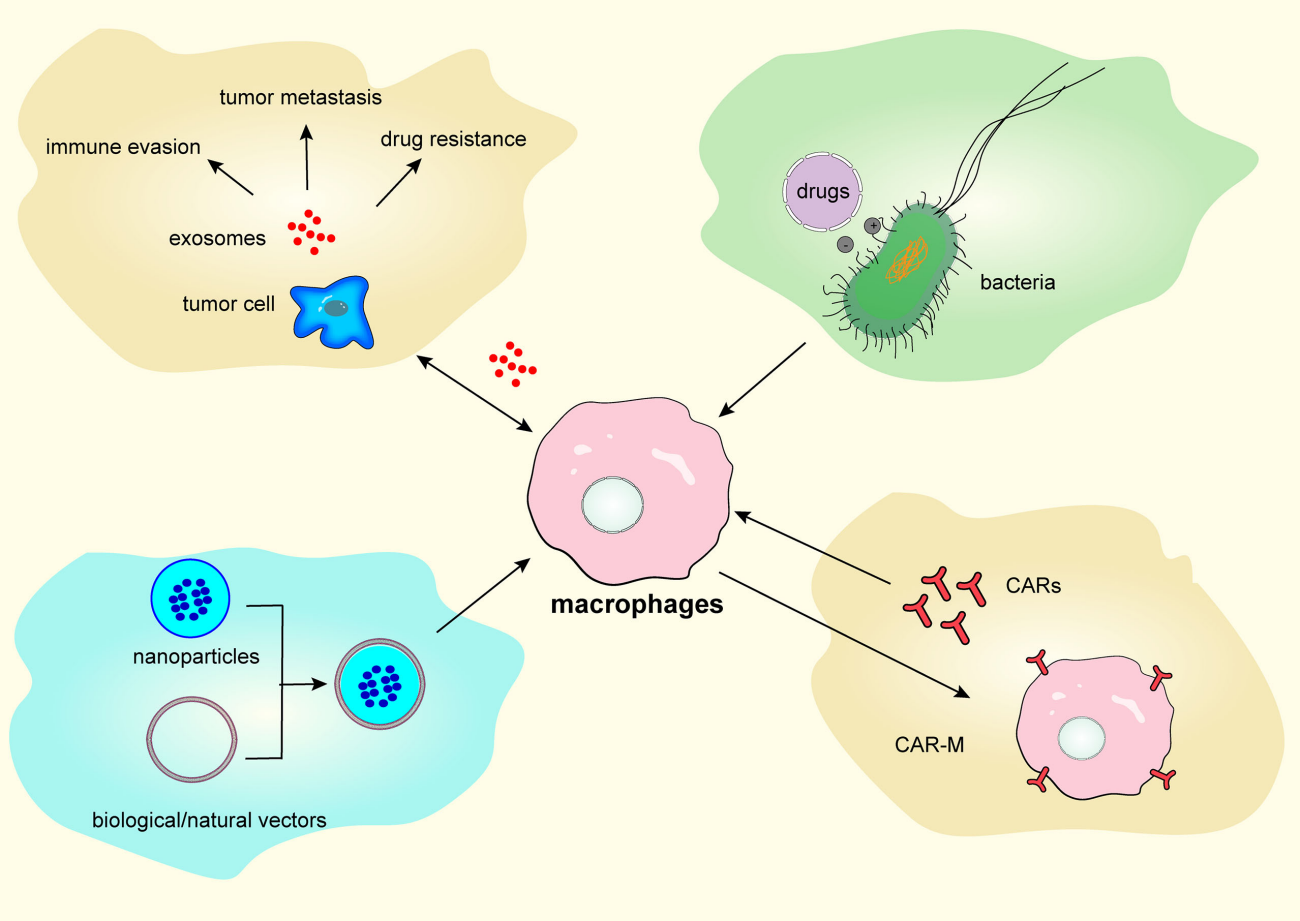
Targeting tumor-associated macrophages repolarization *via* exosomes, bacterial vectors, nanocarriers, and chimeric antigen receptor-macrophage therapy.

In this section, we discuss several approaches for targeting TAM repolarization, including exosomes, bacterial vectors, nanocarriers, and CAR-macrophage (CAR-M) therapy, as shown in **Figure 3**.

#### 4.3.1 Targeting Macrophage-Derived and Cancer Cell-Derived Exosomes

Exosomes are small extracellular vesicles enclosed by a lipid membrane, typically between 30 and 150 nm in diameter, that have been found to participate in the interaction between tumor cells and macrophages. In general, both macrophage-and cancer cell-derived exosomes are associated with tumor immune evasion, metastasis, and drug resistance through various signaling pathways (64). For example, exosomal miR-138-5p derived from breast cancer cells suppresses the expression of KDM6B in macrophages, thereby suppressing M1-related gene expression and promoting breast cancer metastasis (65). Therefore, targeting exosomes derived from macrophages and tumors to eliminate tumors has attracted attention. Improving the ability of exosomes to target tumors and using exosomes to mediate drugs to overcome biological barriers, including the blood–brain barrier and gastrointestinal tract, are emerging as potential therapeutic strategies (66). However, these strategies still lack a comprehensive theoretical understanding and exosome manufacturing remains a challenge.

#### 4.3.2 Bacterial Therapy and Bacterial Carriers

Bacteria and other microbes have been detected in human tumors. A comprehensive analysis of 1,526 tumor samples found that bacterial components are prevalent in human solid tumors, especially breast tumors (67). Bacterial therapy has been widely studied as an important strategy for cancer tumor treatment. For example, some studies showed that ablation of bacteria in pancreatic cancer promotes immune cell infiltration and reduces M2 macrophages in a murine model, while other studies demonstrated that some bacteria are associated with improved clinical outcomes (68). In general, bacteria that cause acute infectious diseases, such as *Salmonella* and Mycobacterium, contribute to M1 polarization. In contrast, bacteria that cause chronic infections are more inclined towards M2 polarization (69). Therefore, cancer treatment utilizing bacteria that can stimulate M1 polarization could be effective, and studies on the use of bacterial treatment to differentiate TAMs from an anti-tumor phenotype already exist. *Listeria monocytogenes*, a bacterium with anti-tumor immune potential, induces immune responses and expresses tumor-associated antigens. The attenuated ΔactA/ΔinlB strain is commonly used for tumor vaccine development, while its ability to enrich antigen-presenting cells has been exploited by researchers to induce repolarization of CD11b+ TAMs and successfully trigger therapeutic anti-tumor immunity (70). Recently, the tumor-targeting ability of bacteria has also been explored to carry therapeutic agents and deliver them precisely into the TME, where they participate in repolarization after being phagocytosed by M2 macrophages (71).

#### 4.3.3 NPs as Drug Carriers

Another emerging strategy for targeting TAMs is the use of NPs, which are focused on overcoming the immunosuppressive TME and *in vivo* delivery barriers. Increased advancements in biomedical NP research has revealed the role of nano-targeted drug delivery systems (nano-TDDS) in enhancing efficacy and improving prognosis. Various nanomaterials have been used to build nano-TDDSs, and substantial progress has been made in this regard. Liposomes, polymeric NPs, and inorganic NPs have all been employed for the development of nano-TDDS and TAM-related tumor therapy. Hot topics of research in this area include, but are not limited to, mimicking biological/natural vectors and metal-organic framework (MOF)-based NPs (72, 73). For instance, lipopolysaccharide-induced macrophage membranes were designed to deliver Fe3O4 NPs and Toll-like receptor 7 agonist imiquimod (R837) (59). Following phagocytosis of PLGA-ION-R837@M by TAMs, Fe3O4 NPs and R837 induced polarization of TAMs to the M1 phenotype *via* IRF5 and NF-κB signaling pathways. The application of this nanocarrier effectively increased the M1/M2 ratio from 0.36 to2.88. Another study linked *Escherichia coli* MG1655 and resiquimod (R848) by electrostatic interactions and used bacterial vectors to target hypoxic tumors, regulating the repolarization of M2 macrophages into M1 macrophages by releasing R848 (71). These two types of nano-TDDS using biological/natural vectors exert anti-tumor effects by increasing M1 macrophage levels to modulate the pro-inflammatory TME. In addition, MOF-NPs have emerged as multifunctional platforms for next-generation drug delivery systems and MOF-bearing immune agonists (isMOF) have been developed to enhance the immune response, followed by surface modification of isMOF NPs with zoledronic acid (74). The application of these NPs, known as BT-isMOF, not only induced high levels of CD86 expression on macrophages, which suggests an M1 phenotype, but also demonstrated potent inhibitory effects against bone metastasis in mice. In summary, although NP-based TAM-targeted therapy has transport barriers, such as low drug solubility and short half-life, it is a promising direction for tumor immunotherapy in the future.

#### 4.3.4 Engineering Macrophages to Act Like M1 Cells—CAR-M Cells

CAR-T cell therapy has made breakthroughs in the treatment of refractory hematological tumors, such as acute lymphoblastic leukemia and diffuse large B-cell lymphoma, but has not been effective in solid tumors (28). Therefore, the development of other immune cells using the CAR platform to treat solid tumors is emerging, and CAR-M technology presents a new immunotherapy strategy. Owing to the large number of macrophages infiltrating the TME, CAR-M technology regulates phagocytosis of macrophages, enhances their antigen-presenting activity, and blocks them in M1 phenotype, thereby improving the immunosuppressive microenvironment (75). Recently, Klichinsky et al. developed a robust gene transfer approach to engineer sustained pro-inflammatory signaling in macrophages in the human TME by delivering first-generation CARs encoding the CD3ζ signaling domain to the human macrophage THP-1 cell line *via* an adenoviral vector (Ad5f35) (76). CAR-M cells have been shown to eradicate tumor cells and reduce the metastatic tumor burden in a dose- and time-dependent manner in *in vivo* humanized mouse models. The advantage of this new cell-editing approach is that CAR serves as a platform for precise targeting of antigen-expressing tumor cells, overcoming the difficulty of reaching the TME, while the edited macrophages are able to maintain an anti-tumor phenotype.

## 5 Conclusion

TAMs located in the TME have the following characteristics: 1) TAMs have an M2-like macrophage phenotype and can exert anti-inflammatory and pro-tumor effects; 2) studies have shown that TAMs decrease the efficacy of ICB and CAR-T cell therapy; 3) TAM polarization is regulated by the various signaling pathways and regulating these pathways can effectively alter TAM phenotype; and 4) strategies targeting TAM repolarization, such as exosomes, bacterial therapy, NPs, and CAR-M therapy, show potential in the treatment of solid tumors. Paradigm-shifting discoveries of targeted TAM polarization in tumor immunotherapy and their remarkable effect on some tumors have made it a hot research topic. Nevertheless, almost all studies have been conducted at the preclinical stage. It is important to acknowledge that most drugs targeting TAMs still face difficulties, such as transport barriers, complex preparation methods, and unstable drug forms. In conclusion, research on TAM repolarization is still in the preliminary stages and several different targeting approaches are under investigation. The efficacy of these approaches in combination with other anti-tumor strategies in different tumors warrants further investigation.

## Author Contributions

JG, YL, and LW had a substantial contribution to the conception of the work, drafted the work, revised it critically and approved it for publication. All authors contributed to the article and approved the submitted version.

## Funding

This work was supported by grants from the National Natural Science Foundation of China (grant No. 81873450, 82170181), and Beijing Hospitals Authority Youth Programme (code: QML20200201) to LW.

## Conflict of Interest

The authors declare that the research was conducted in the absence of any commercial or financial relationships that could be construed as a potential conflict of interest.

## Publisher’s Note

All claims expressed in this article are solely those of the authors and do not necessarily represent those of their affiliated organizations, or those of the publisher, the editors and the reviewers. Any product that may be evaluated in this article, or claim that may be made by its manufacturer, is not guaranteed or endorsed by the publisher.
